# The novel roles of YULINK in the migration, proliferation and glycolysis of pulmonary arterial smooth muscle cells: implications for pulmonary arterial hypertension

**DOI:** 10.1186/s40659-023-00480-z

**Published:** 2023-12-07

**Authors:** Yi-Chia Wu, Wei-Ting Wang, Ming-Chun Yang, Yu-Tsun Su, Jwu-Lai Yeh, Jong-Hau Hsu, Jiunn-Ren Wu

**Affiliations:** 1 Division of Plastic Surgery, Department of Surgery, Kaohsiung Medical University Hospital, Kaohsiung Medical University, Kaohsiung, 80708 Taiwan; 2 Department of Plastic Surgery, Kaohsiung Municipal Ta-Tung Hospital, Kaohsiung, 80145 Taiwan; 3 Department of Surgery, School of Medicine, Kaohsiung Medical University, Kaohsiung, 80708 Taiwan; 4 Regenerative Medicine and Cell Therapy Research Center, Kaohsiung Medical University, Kaohsiung, 80708 Taiwan; 5 Department of Pediatrics, E-Da Hospital/I-Shou University, No. 1, Yi-Da Road, Jiao-Su Village, Yan-Chao District, Kaohsiung, 82445 Taiwan; 6 Department of Pediatrics, School of Medicine, College of Medicine, I-Shou University, Kaohsiung, Taiwan; 7 Department of Pharmacology, School of Medicine, College of Medicine, Kaohsiung Medical University, Kaohsiung, Taiwan; 8 Division of Pediatric Cardio-Pulmonology, Department of Pediatrics, Kaohsiung Medical University Hospital, Kaohsiung Medical University, Kaohsiung, Taiwan; 9 Department of Pediatrics, School of Medicine, College of Medicine, Kaohsiung Medical University, Kaohsiung, Taiwan

**Keywords:** YULINK, Pulmonary arterial hypertension (PAH), Pulmonary arterial smooth muscle cells (PASMCs), Migration, Proliferation, Glycolysis

## Abstract

**Background:**

Abnormal remodeling of the pulmonary vasculature, characterized by the proliferation and migration of pulmonary arterial smooth muscle cells (PASMCs) along with dysregulated glycolysis, is a pathognomonic feature of pulmonary arterial hypertension (PAH). *YULINK* (*MIOS*, Entrez Gene: 54468), a newly identified gene, has been recently shown to possess pleiotropic physiologic functions. This study aims to determine novel roles of YULINK in the regulation of PAH-related pathogenesis, including PASMC migration, proliferation and glycolysis.

**Results:**

Our results utilized two PAH-related cell models: PASMCs treated with platelet-derived growth factor (PDGF) and PASMCs harvested from monocrotaline (MCT)-induced PAH rats (PAH-PASMCs). *YULINK* modulation, either by knockdown or overexpression, was found to influence PASMC migration and proliferation in both models. Additionally, YULINK was implicated in glycolytic processes, impacting glucose uptake, glucose transporter 1 (GLUT1) expression, hexokinase II (HK-2) expression, and pyruvate production in PASMCs. Notably, YULINK and GLUT1 were observed to colocalize on PASMC membranes under PAH-related pathogenic conditions. Indeed, increased YULINK expression was also detected in the pulmonary artery of human PAH specimen. Furthermore, YULINK inhibition led to the suppression of platelet-derived growth factor receptor (PDGFR) and the phosphorylation of focal adhesion kinase (FAK), phosphoinositide 3-kinase (PI3K), and protein kinase B (AKT) in both cell models. These findings suggest that the effects of YULINK are potentially mediated through the PI3K-AKT signaling pathway.

**Conclusions:**

Our findings indicate that YULINK appears to play a crucial role in the migration, proliferation, and glycolysis in PASMCs and therefore positioning it as a novel promising therapeutic target for PAH.

**Supplementary Information:**

The online version contains supplementary material available at 10.1186/s40659-023-00480-z.

## Background

Pulmonary arterial hypertension (PAH) is an unusual but life-threatening disease characterized by increases in pulmonary vascular resistance (PVR), which eventually leads to right ventricular (RV) failure and death [1]. In the 6th World Symposium on Pulmonary Hypertension in Nice, France (2018), one of the new definitions of PAH was redefined as a mean pulmonary artery pressure (mPAP) > 20 mmHg, pulmonary arterial wedge pressure > 15 mmHg, plus pulmonary vascular resistance index > 3 Wood units/m^2^ [2]. The estimated prevalence of PAH was reported to be 26–33 cases per million in the US and 20–40 cases per million in Europe [3].

In PAH, continuously elevated mPAP has been demonstrated to correlate with the reconstruction and remodeling of pulmonary vascular structure characterized by an accumulation of different vascular cells in endothelial proliferation and hyperplasia of the medial muscular layer and collagen-rich fibrosis of adventitia. Excessive pulmonary artery smooth muscle cell (PASMC) proliferation and migration resulting in pulmonary arterial wall thickening is the most conspicuous feature in the pathological development of PAH [4]. Among these factors, platelet-derived growth factor (PDGF) has been widely recognized as a key regulator that triggers uncontrolled and dysregulated migration and proliferation of PASMCs in PAH pathogenesis [5].

In addition to the pathophysiological hyperproliferation and cell migration of PASMCs, a cancer-like abnormal metabolic phenotype named the “Warburg effect” has been clarified in PAH [6]. In this metabolic abnormality, the cells tend to favor an anaerobic respiration pathway with upregulated glycolysis over defective mitochondrial respiration, and furthermore, PDGF-triggered PASMC proliferation is evidenced as accompanying higher glucose consumption and lactose production. Treatment with PDGF stimulates a constitutive increase in glucose transporters and the expression of the glycolysis marker GLUT1 [7, 8]. Upregulated glycolytic gene expression was also found in hyperproliferative pulmonary artery fibroblasts isolated from PAH patients [9]; therefore, targeting PDGF-related signaling pathways has been considered an attractive strategy in PAH therapeutics.

According to comparative evolutionary genomics analysis, we previously identified a gene named *YULINK* (*MIOS*, Entrez Gene: 54468) that is evolutionarily conserved from zebrafish to humans and encodes an 875 amino acid protein containing WD40 repeats at the N-terminus in humans [10]. Deficiency of YULINK resulted in irregular Ca^2+^ cycling and contributed to the induction of cardiac arrhythmia in both mouse and human models [10]. YULINK contains a highly conserved domain, MIOS (missing oocyte, gene ID: 33399), which was revealed to participate in the regulation of mTOR signaling [11]. mTOR signaling plays critical roles in cell migration, cell proliferation and energy homeostasis in PASMCs in PAH [12]; however, whether YULINK participates in PAH-related pathophysiological abnormalities, especially in cell migration, proliferation and glycolytic function, has not yet been investigated.

In this study, the role of YULINK in cell migration, proliferation and glycolysis in PASMCs treated with PDGF or PASMCs derived from rats with MCT-induced PAH was examined, and the results indicated that the level of YULINK expression was correlated with cell migration and proliferation in PASMCs treated with PDGF or PASMCs derived from rats with MCT-induced PAH (PAH-PASMCs). Glucose uptake and glycolysis in PASMCs could be regulated by YULINK through GLUT1 interaction. YULINK suppression inhibited cell migration, proliferation, and glycolysis in PAH cell models, which could be mediated through the PI3K-AKT signaling pathways. These findings provide a possible mechanistic link between YULINK and the pathogenesis of PAH.

## Materials and methods

### Animals

The protocol of the animal study performed in this research was approved by the Animal Care and Use Committee of Kaohsiung Medical University (IACUC-110029). Adult male Sprague‒Dawley rats weighing approximately 250 g were provided by BioLASCO Taiwan (Taipei, Taiwan) and raised under a constant temperature and controlled illumination.

### PASMC isolation and MCT-induced PAH-PASMCs

For PASMC isolation, the rats were sacrificed by anesthesia with an overdose of isoflurane, and all efforts were made to reduce their suffering. The skin of the rats was sterilized with 75% ethanol, and the chests were opened under sterile conditions. Then, the hearts and lungs were removed, and the pulmonary arteries were dissected. The PASMCs were then isolated as described previously [13]. PASMCs were cultured with Dulbecco’s modified Eagle’s medium (DMEM, 11966-025, Gibco, MA, U.S.A.) supplemented with 10% fetal bovine serum (FBS) and 1% antibiotic-antimycotic, 100× (15140122, Thermo Fisher Scientific, MA, U.S.A.) under 5% CO_2_ at 37 °C. The PASMCs were examined by immunofluorescence staining to confirm their purity. Over 95% of the cells were composed of PASMCs. To obtain PAH-PASMCs from the animal disease model, a single injection of MCT (60 mg kg^−1^) was administered to the rats via intraperitoneal injection for 4 weeks to develop PAH as described previously [13], and the rats were then sacrificed for PASMC isolation for further experiments.

### YULINK knockdown

Both the pGIPZ lentiviral shRNAmir vector packaged with a short hairpin RNA targeting *YULINK* (Clone ID. V3LHS_374795 and gene access no. NM_019005, respectively) and a nontargeting scramble control shRNA vector RHS4346 were purchased from Open Biosystems (AL, U.S.A.). PASMCs were infected with the viruses with 8 µg/ml polybrene (MilliporeSigma, MA, U.S.A.) for 72 h and selected by using puromycin (2 µg/ml) to generate gene knockdown. Cells with *YULINK* knockdown were then maintained with media containing antibiotics.

### YULINK overexpression

To generate *YULINK* overexpression, the full-length coding region of *YULINK* was constructed into a lentiviral pCDH-CMV-MCS-EF1α-copGFP vector (System Biosciences, CA, U.S.A.) and applied to PASMCs with 8 µg/ml polybrene for 72 h. Puromycin (2 µg/ml) was used to select the cells that were successfully infected. Selected PASMCs with *YULINK* overexpression were maintained in media containing antibiotics.

### Transwell migration assay

A Transwell migration assay [14] was used to investigate PDGF-triggered and PAH-related cell migration. Briefly, PASMCs were seeded into the upper compartment of Transwell chambers (3413, Corning Inc., NY, U.S.A.) with DMEM containing 2% FBS. The medium in the lower compartment contained 10% FBS with or without various doses of PDGF (5 ng/ml, 20 ng/ml). After 24 h, the cells were washed with PBS and fixed with 4% paraformaldehyde. The fixed cells were stained with crystal violet. The non-migrated cells were wiped off by using cotton swabs. The migrated PASMCs were then recorded and photographed through a microscope.

### PASMC proliferation and viability analysis

MTT (3-(4,5-dimethylthiazol-2-yl)-2,5-diphenyltetrazolium bromide) was used to examine cell proliferation. After the treatments, PASMCs were washed with PBS, and the culture medium was replaced with fresh medium containing 10 µg/ml MTT for 1 h. The purple-colored formazan crystals produced by living cells were dissolved in dimethyl sulfoxide (DMSO). Cell viability was then quantified by measuring the absorbance at a wavelength of 570 nm.

### Western blot

As described previously [15], cell lysates were prepared and fractionated using sodium dodecyl sulfate–polyacrylamide gel electrophoresis (SDS-PAGE) and blotted with antibodies. The Western Lightning® Plus-ECL detection system (NEL104001EA, PerkinElmer, MA, U.S.A.) was used to determine protein expression. The primary antibodies used were as follows: Mios (ab202274, Abcam, Cambridgeshire, U.K.), p-FAK Y397 (ab81298, Abcam), FAK (ab40794, Abcam), GLUT1 (73015, Cell Signaling, MA, U.S.A.), hexokinase II (2867, Cell Signaling), PDGFR (ab203491, Abcam), p-PI3k p85 Y458 + p55 Y199 (ab278545, Abcam), PI3k p85 (4292S, Cell Signaling), p-AKT S473 (4060, Cell Signaling), p-AKT T308 (13038, Cell Signaling), AKT (9272, Cell Signaling), and β-actin (3700, Cell Signaling). The secondary antibodies used were anti-rabbit IgG (7074, Cell Signaling) and anti-mouse IgG (7076, Cell Signaling). The Western blot results were further determined using ImageJ software and normalized with an internal control.

### Flow cytometry analysis

After the treatments, both floating dead and attached living cells were collected and fixed with 70% ethanol to increase the permeability for later propidium iodide (PI) staining. Fixed cells were washed three times with PBS to remove ethanol before staining with PI. Meanwhile, RNaseA was used to eliminate any possibility of RNA interference. The cell cycle profile was analyzed by using an LSR II Flow Cytometer (BD Biosciences) and FlowJo 7.6 software (FlowJo LLC, OR, U.S.A.).

### Glucose uptake analysis

In this study, glucose uptake was analyzed by using a Fluorometric 2-Deoxyglucose Assay Kit (KA3751, Abnova, Taipei, Taiwan). PASMCs (2.5 × 10^4^ cells/well) were seeded into a 24-well plate overnight to allow attachment. On the day of examination, 10% FBS regular culture medium was replaced with 0.5% FBS culture medium for a 1-hour starvation. Starved cells were then washed three times with PBS and treated with 10 mM 2-DG glucose uptake mix for 20 min. Afterwards, the cells were washed three times with PBS to remove exogenous 2-DG and lysed with 90 µl of extraction buffer. Cell lysates were cooled on ice for 5 min and neutralized with 10 µl of neutralization buffer followed by mixing with assay buffer at 37 °C for 40 min. The level of glucose uptake was determined by measuring fluorescence at Ex/Em = 535/587 nm.

### Immunofluorescence assay

Cells were seeded on glass coverslips for the treatment. Afterwards, the cells were washed with PBS and fixed with 3% buffered paraformaldehyde for immunostaining. Fluorescence images of stained cells were recorded using a Zeiss Axioplan 2 Upright Fluorescence Microscope and analyzed with Volocity 3.6.1 software (Improvision, West Midlands, U.K.). The primary antibodies were MIOS (ab122822, Abcam) and GLUT1 (ab40084, Abcam); the secondary antibodies were goat anti-rabbit IgG (H + L), F(ab′)2 fragment, CF^TM^568 (SAB4600400, Merck, Darmstadt, Germany), goat anti-mouse IgG (H+L), highly cross-adsorbed (min X Rat), CF^TM^568 (SAB4600311, Merck), and goat anti-rabbit IgG (H+L), highly cross-adsorbed, CF^TM^488 (SAB4600045, Merck). DAPI (4′,6-diamidino-2-phenylindole) was used to stain the nuclei.

### Glycolytic function analysis

During the process of glycolysis, glucose is catalyzed by enzymes and finally converted into pyruvic acid. In this research, we determined pyruvate production by using the Pyruvate Assay Kit (ab65342, Abcam) following the manufacturer’s guidelines to examine the correlation between the level of YULINK and glycolysis in PASMCs. Briefly, 8 × 10^4^ PASMCs with varying YULINK levels were seeded into a 96-well plate and treated with or without PDGF for 48 h, followed by measurement of pyruvate concentrations. The content of pyruvate was determined by measuring the fluorescence at Ex/Em = 540/590 nm, which was generated by pyruvate oxidase via enzyme reactions.

### Histological analysis

The procedures of the sample preparation were described previously [16]. Briefly, the bronchial artery tissue was fixed with 4% paraformaldehyde and embedded in paraffin wax before sectioning to 4 μm-thick specimens for immunohistochemistry (IHC). Samples were deparaffinized and rehydrated with 100%, 95%, and 70% alcohol before being washed with distilled water. The samples were then stained with aniline blue solution and differentiated with 1% acetic acid solution. After incubation, the samples were washed with distilled water followed by dehydration with 95% and absolute ethyl alcohol. Finally, the samples were mounted with resinous mounting medium for later microscopy. Furthermore, we acquired a human right pulmonary artery specimen of an individual diagnosed with severe PAH, from Kaohsiung Medical University Hospital Biobank (approved by the Institutional Review Board of Kaohsiung Medical University Hospital, KMUHIRB-E(I)-20230233) to assess YULINK expression in a clinical context. A normal pulmonary artery tissue was used as a control for comparative analysis.

For immunohistochemical analysis of increased levels of YULINK, YULINK (rabbit anti-MIOS, ab122822, Abcam) (1:100) antibody was selected for immunostaining. The images were recorded and analyzed using Motic EasyScan One (Motic, Xiamen, China) and Motic DSAssistant software.

### Membrane protein extractions

A Mem-PER™ Plus Membrane Protein Extraction Kit (89842, Thermo Fisher Scientific) was used for membrane protein extraction. Cells were resuspended and collected for centrifugation at 300×*g* for 5 min. Cell pellets were further washed with Cell Wash Solution followed by another 5-min centrifugation at 300×*g*. Permeabilization buffer was added to the pellets for a 10-min incubation at 4 °C with constant mixing. Permeabilized cells were then centrifuged at 16,000×*g* for 10 min to remove cytosolic fractions. Solubilization buffer was added to the samples for a 30-min incubation at 4 °C with constant mixing. Membrane proteins were finally isolated and extracted after a 15-min centrifugation at 16,000×*g* at 4 °C.

### Immunoprecipitation analysis

The immunoprecipitation analysis was conducted using the Pierce Crosslink Magnetic IP/Co-IP Kit (Thermo Fisher Scientific, 88805) in accordance with the manufacturer’s instructions. Prior to antigen immunoprecipitation, the YULINK antibodies were crosslinked to magnetic beads using DSS. Cell protein lysates, prepared in advance, were then incubated with the antibody-crosslinked beads at room temperature to facilitate the elution of bound antigens.

### Statistical analysis

SPSS 17.0 (one-way ANOVA followed by post hoc analysis with the Bonferroni correction; SPSS Inc., IL, U.S.A.) was used for statistical analysis. All represented results in this study are derived from at least three independent repeats. Values are presented as the mean ± S.D. A p value < 0.05 indicates a statistical significance between compared groups.

## Results

### Positive correlation between YULINK expression and cell migration in PASMCs

**Fig. 1:**
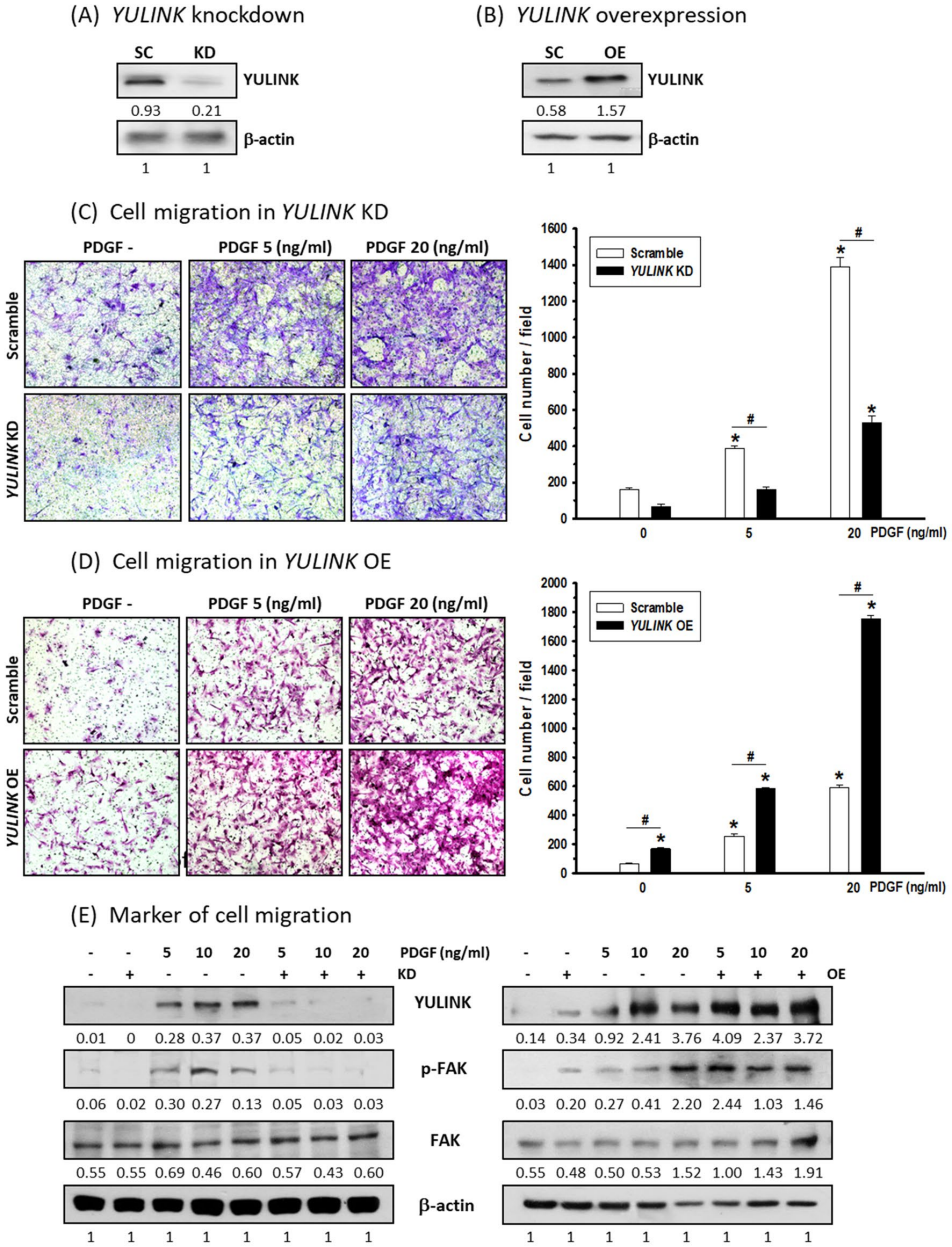
Positive correlation between YULINK expression and cell migration in PASMCs. YULINK expression in PASMCs with **A** *YULINK* knockdown (KD) or **B** overexpression (OE) was examined by western blot analysis. SC stands for scramble control. The numbers labeled below the respective blot lanes indicate the relative fold normalized with the internal control. **C**, **D** Micrographs (magnification 200×) together with bar graphs depict PDGF (5 and 20 ng/ml)-triggered cell migration in PASMCs with **C** *YULINK* KD or **D** *YULINK* OE using Transwell analysis. The values represent the mean of three independent experiments ± standard deviation. *p < 0.05 compared to scramble control without PDGF. ^#^p < 0.05 between compared groups. **E** PASMCs with or without *YULINK* KD or OE were treated with PDGF in a dose-dependent manner (0, 5, and 20 ng/ml) for 24 h and subjected to western blot analysis for the indicated proteins. β-actin served as an internal control

To evaluate the role of YULINK in PASMC migration in either *YULINK* knockdown (KD) or overexpression (OE), PASMCs were analyzed by Western blot analysis, with the efficiency shown in Fig. 1A, B, respectively. To further examine whether diminished YULINK expression could inhibit PASMC migration, PASMCs with or without *YULINK* knockdown were seeded on the permeable upper layer of the cell culture with a dose-dependent PDGF (0, 5, 20 ng/ml) treatment in the culture medium on the bottom layer of the well for cell migration analysis. As shown in Fig. 1C, PASMC migration increased in a dose-dependent manner. Cell migration was significantly reduced in YULINK-deficient PASMCs treated with PDGF, while in contrast, cell migration was enhanced in PASMCs overexpressing *YULINK* with or without PDGF treatment (Fig. 1D).

Furthermore, focal adhesion kinase (FAK), a protein that is well recognized as a marker of cell migration, was also determined by Western blot analysis. In addition, FAK is widely expressed as a cytoplasmic protein tyrosine kinase and plays an important key step in cell migration when activated by phosphorylation at Tyr397 [17]. As shown in Fig. 1E, the administration of PDGF induced either YULINK or FAK phosphorylation at Tyr397 in a dose-dependent manner; however, this PDGF-triggered enhancement of FAK phosphorylation was inhibited in PASMCs with *YULINK* knockdown. In contrast, FAK Tyr397 phosphorylation in response to PDGF treatment was enhanced in PASMCs overexpressing *YULINK* (Fig. 1E). These data illustrate that YULINK could be a positive regulator in PASMCs under PDGF treatment.

### Positive correlation between YULINK expression and cell proliferation in PASMCs

**Fig. 2:**
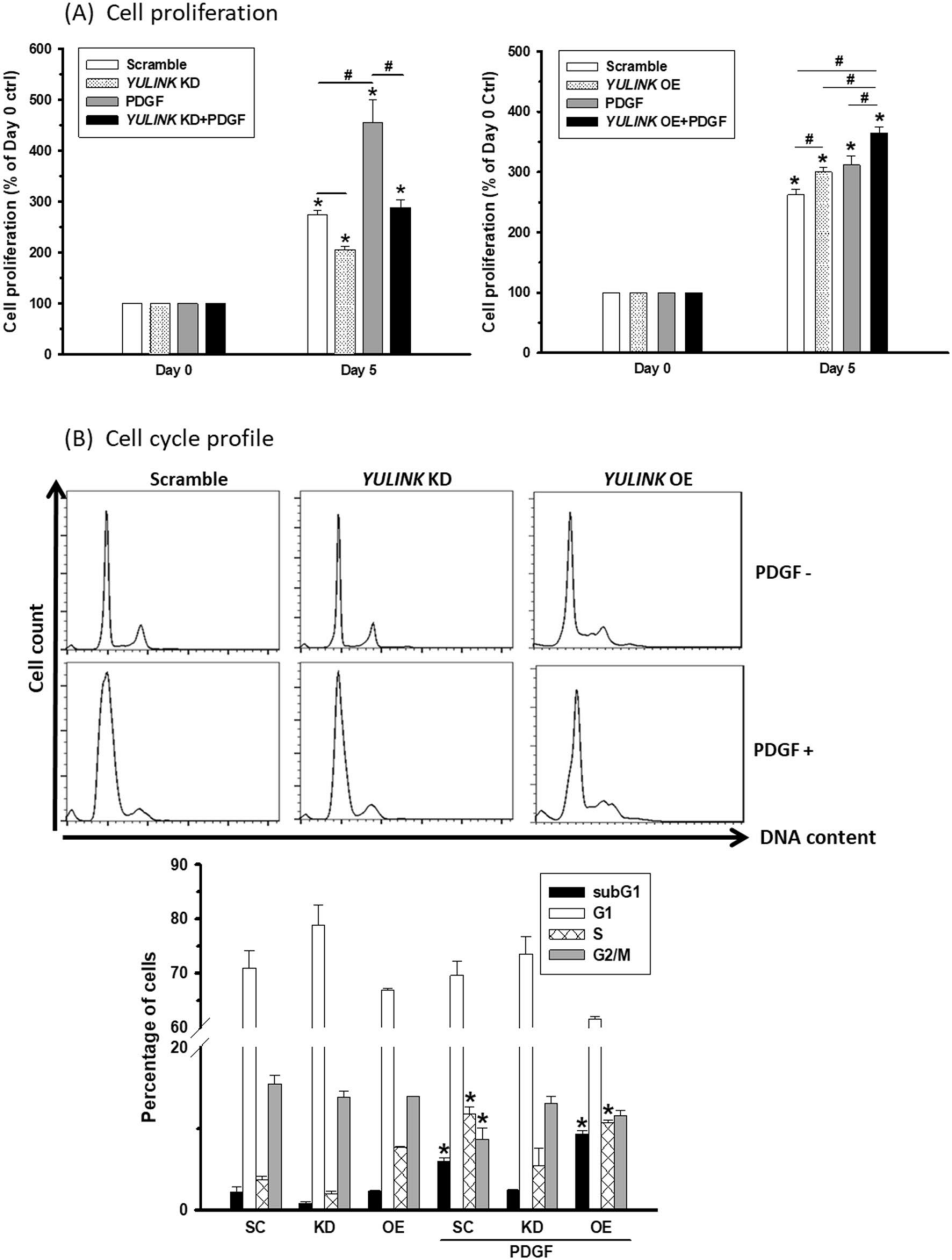
Positive correlation between YULINK expression and cell proliferation in PASMCs. **A** PASMCs with *YULINK* KD or OE were treated with or without PDGF (20 ng/ml) for 5 days before harvest for MTT analysis. **B** PASMCs with the same treatment for 24 h were stained with PI and subjected to flow cytometry. The cell cycle profiles are presented with bar graphs indicating the distributions of the cells in the cell cycle. The values represent the mean of three independent experiments ± standard deviation. *p < 0.05 compared to scramble control without PDGF. ^#^p < 0.05 between compared groups

To investigate whether the YULINK expression level could affect cell proliferation, PASMCs with *YULINK* knockdown (KD) or overexpression (OE) in response to PDGF (20 ng/ml) at 0 and 5 days were subjected to MTT proliferation/viability analysis (Fig. 2A), with increased cell proliferation being noted in PASMCs with PDGF for 5 days compared to those without PDGF treatment. Cell proliferation was inhibited in PASMCs with *YULINK* KD with or without PDGF treatment. In contrast, cell proliferation was enhanced in PASMCs with *YULINK* OE with or without PDGF treatment. Similarly, the colony formation assay provided additional evidence of the relationship between YULINK expression and cell proliferation. In the presence of PDGF treatment or in PAH-PASMCs, numerous and distinct colonies formed. However, when YULINK was preemptively suppressed, the colony formations were noticeably reduced (Additional file 1: Fig. S1).

As shown in Fig. 2B, these results were further confirmed by cell cycle profile analysis. Both floating and attached cells were collected and stained with propidium iodide (PI) for DNA content detection. In comparison to the scramble control, treatment with PDGF triggered a significant increase in the S-phase population, indicating further DNA synthesis for cell proliferation; however, this upregulated S-phase population was not found in PASMCs with *YULINK* knockdown. Even without PDGF treatment, PASMCs with *YULINK* knockdown or overexpression alone slightly suppressed or increased the S-phase population, respectively, accompanied by an increased or decreased G1 phase population, respectively.

Furthermore, existing researches have indicated that PAH pathogenesis involves genomic instability, the production of free radicals, and elevated levels of DNA damage [13, 18], which can lead to apoptosis. However, this effect is likely transient, as cells eventually tend to exhibit increased proliferation during PAH pathogenesis. It is evident that in PASMCs treated with PDGF, there was an increase in the subG1 population, which was further elevated in cells with YULINK overexpression. However, when YULINK was inhibited, this elevation in the subG1 population was significantly mitigated. These results align with the notion that YULINK overexpression, particularly in conjunction with PDGF treatment, leads to genome instability and cell death associated with PAH. This finding also suggests that the inhibition of YULINK could be advantageous in suppressing the pathogenesis of PAH. Taken together, the cell proliferation of PASMCs was positively correlated with YULINK under PDGF treatment.

### YULINK colocalized with GLUT1 and participated in PDGF-triggered glucose uptake and glycolysis

**Fig. 3:**
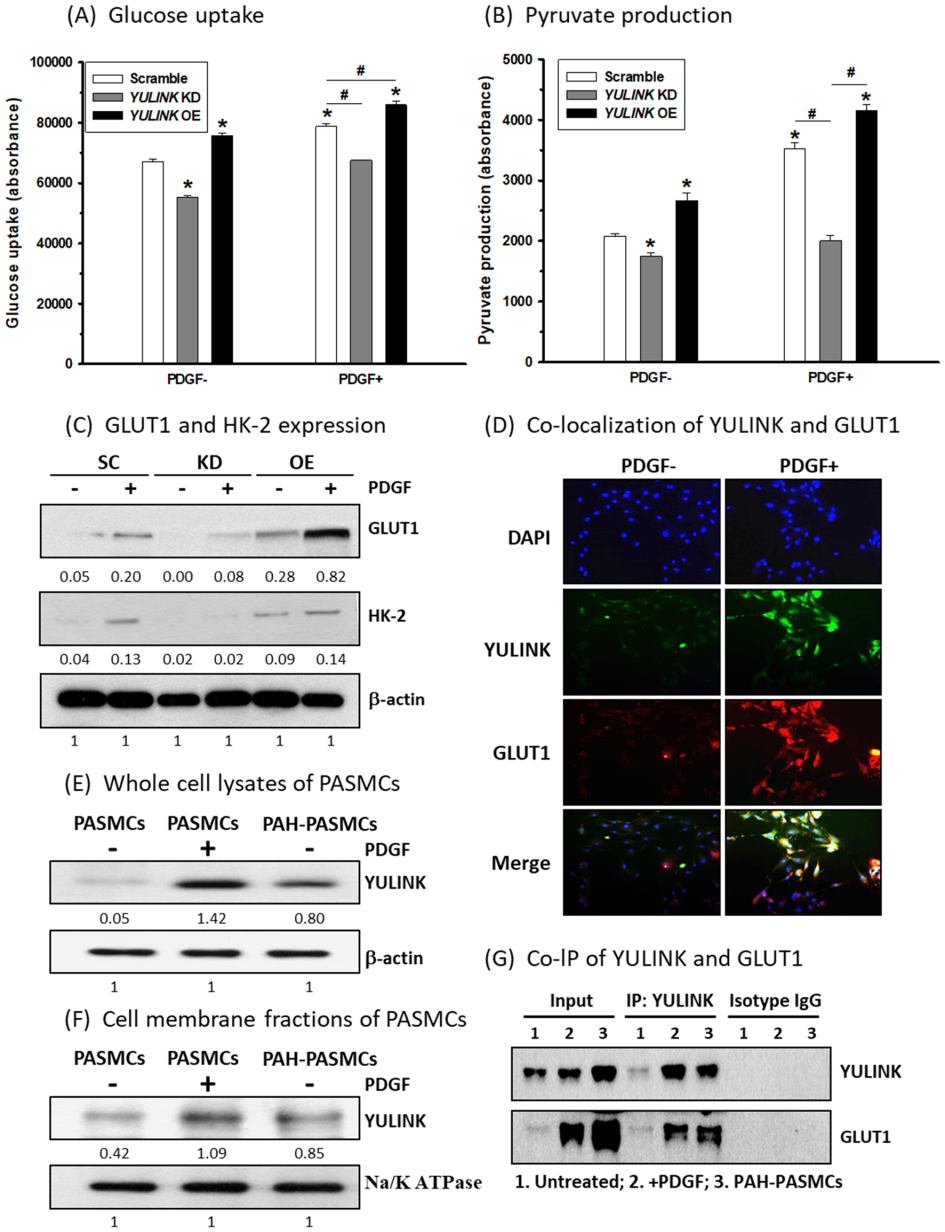
YULINK interacted with GLUT1 and participated in PDGF-triggered glucose uptake and glycolysis. The bar graphs illustrate **A** glucose uptake and **B** pyruvate production in PASMCs with *YULINK* KD or OE treated with or without PDGF (20 ng/ml) for 24 h. The values represent the mean of three independent experiments ± standard deviation. *p < 0.05 compared to scramble control without PDGF. ^#^p < 0.05 between compared groups. **C** Western blot analysis indicates the expression of GLUT1 and HK-2 in normal PASMCs, PASMCs with *YULINK* KD, and PASMCs with *YULINK* OE treated with or without PDGF for 24 h. The numbers labeled below the respective blot lanes indicate the relative fold normalized with the internal control. **D** Representative images from immunofluorescence analysis indicate the expression of YULINK (green) and GLUT1 (red), and DAPI (blue) indicates nuclear staining. Magnification 200×. **E** Whole-cell lysates and **F** membrane fractions derived from PASMCs or PAH-PASMCs with or without 20 ng/ml PDGF treatment and PAH-PASMCs were subjected to Western blot analysis for YULINK expression. β-Actin and Na/K ATPase served as internal controls. **G** Membrane protein lysates obtained from PASMCs subjected to indicated treatments were used for immunoprecipitation with YULINK antibody-conjugated beads. The proteins pulled down were subsequently analyzed via Western blot to assess the expression of YULINK and GLUT1. While Input serves as positive control, Isotype IgG serves as negative control

Increased glucose uptake and glycolysis in both preclinical studies and PAH patients were noted in hyperproliferative PASMCs, which have increased GLUT1 1 expression [19–21]. Therefore, one of our goals was to investigate whether YULINK expression was related to either glucose uptake or glycolysis in PASMCs. PASMCs were treated with the fluorescent glucose analog 2-deoxy-d-glucose (2-DG) to determine the levels of glucose uptake in response to PDGF treatment. Since the 2-hydroxyl group of 2-DG is replaced by hydrogen, it would be transported and thereby accumulate in cells without entering glycolysis, so the fluorescence of accumulated 2-DG would indicate the level of glucose uptake. As shown in Fig. 3A, glucose uptake was inhibited in PASMCs with *YULINK* knockdown compared to the scramble control. In contrast, glucose uptake was enhanced in PASMCs with *YULINK* overexpression, while PDGF administration promoted glucose uptake in PASMCs, which was inhibited in PASMCs with *YULINK* knockdown. On the other hand, glucose uptake was further enhanced in PASMCs overexpressing *YULINK* under PDGF treatment (Fig. 3A).

We next examined whether YULINK expression was correlated with glycolysis in PASMCs. PASMCs with *YULINK* knockdown or overexpression were collected for pyruvate production under PDGF treatment for 48 h, and as shown in Fig. 3B, pyruvate production was significantly increased in PASMCs under PDGF treatment, which indicated an upregulation of glycolysis. Furthermore, pyruvate production was further enhanced in PASMCs with *YULINK* overexpression under PDGF treatment, but in contrast, pyruvate production was inhibited in either PDGF-treated or no-PDGF-treated PASMCs with *YULINK* knockdown.

Since glucose transporter 1 (GLUT1), the major member of the Glut family, was reported to be expressed in vascular smooth muscle cells and facilitate glucose uptake for vascular contractility and reactivity [22], we next examined whether the alteration of YULINK expression further affected GLUT1 expression. Consistently, while *YULINK* knockdown decreased GLUT1 expression in PASMCs, the level of GLUT1 was increased with *YULINK* overexpression regardless of the presence or absence of PDGF treatment (Fig. 3C). Additionally, the level of hexokinase II (HK-2), the key catalytic kinase that converts glucose to glucose-6-phosphate in the process of glycolysis, was affected in the same pattern as GLUT1 by western blotting analysis (Fig. 3C).

To investigate whether YULINK and GLUT1 had protein interactions under PDGF treatment, immunofluorescent analysis was performed for PASMCs with or without PDGF treatment. As shown in Fig. 3D, GLUT1 and YULINK were not only increased but also colocalized in PASMCs treated with PDGF. Taken together, YULINK expression could regulate glucose uptake and glycolysis through GLUT1 protein interaction under PDGF treatment, thereby implying a possible role of YULINK in PAH-related pathogenesis.

In addition to PDGF-treated PASMCs, PASMCs derived from monocrotaline-induced PAH rat models were also examined. As shown in Additional file 1: Fig. S2, medial hypertrophy was noted by hematoxylin and eosin (H & E) staining of the pulmonary artery in the MCT-induced PAH group, confirming the efficacy of the MCT treatment. Since GLUT1 is known to localize on the cell membrane and functions as a glucose transporter, we conducted further investigations to determine whether YULINK also exhibits an increase in cell membrane localization in PASMCs during PAH pathogenesis. As shown in Fig. 3E, F, increased YULINK expression was noted in both whole-cell lysates and membrane fractions in PASMCs under PDGF treatment and in PAH-PASMCs. A co-immunoprecipitation analysis provided further evidence of the colocalization of YULINK and GLUT1 on the cell membrane in PASMCs. Under the conditions related to PAH pathogenesis, it was evident that GLUT1 was notably present in the protein extracts that were pulled down along with YULINK (Fig. 3G). These findings suggested that YULINK could be increased in the cell membrane of PASMCs for protein interaction with GLUT1 during PAH pathological development.

### Enhanced YULINK expression in MCT-induced PAH rats and a clinical PAH patient

**Fig. 4:**
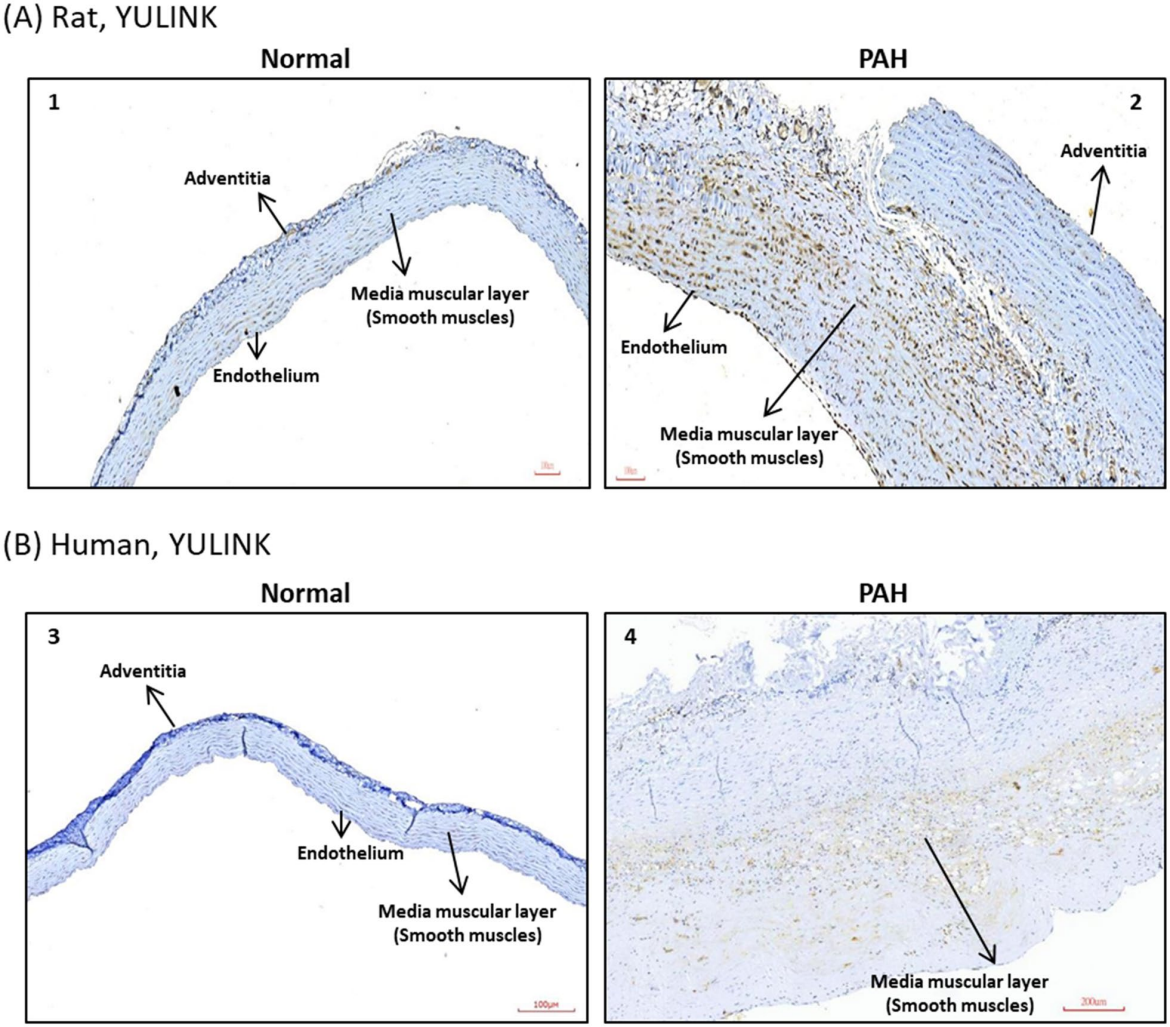
Enhanced YULINK expression in MCT-induced PAH rats and human PAH specimen. **A** Pulmonary artery tissues were derived from normal and MCT-induced PAH rats for IHC staining. The brown color in the photomicrograph indicates the expression of YULINK expression. **B** Tissue sample from the right pulmonary artery of a clinical patient with severe PAH was subjected to IHC staining to assess YULINK expression. A normal pulmonary artery tissue was used as a control for comparison. Scale bar in 1–3: 100 µM, and 4: 200 µM

How YULINK is expressed in MCT-induced rats in vivo was next examined. Immunohistochemical (IHC) staining of the pulmonary artery in the MCT-induced PAH group revealed increased brown staining in the thickening vasculature of the pulmonary artery compared to the normal control. In normal animals, YULINK expression is minimal within the pulmonary artery. However, following PAH induction, there is a significant increase in the expression of YULINK, and it is distributed more extensively throughout the tissue including in media muscular layer (Fig. 4A), which indicated increased YULINK expression in the pulmonary artery of the MCT-induced PAH rat model.

In addition to the animal model, we obtained a human right pulmonary artery specimen of an individual with severe PAH and performed immunohistochemistry (IHC) staining to analyze YULINK expression in an actual clinical case. Consistent with the results observed in rat tissue, our findings in the human specimen also demonstrate a significant increase in YULINK expression particularly in media muscular layer in the human PAH specimen compared to the control from a normal pulmonary artery specimen (Fig. 4B).

### YULINK suppression inhibited the migration and proliferation of PASMCs derived from an MCT-induced PAH rat model (PAH-PASMCs)

**Fig. 5:**
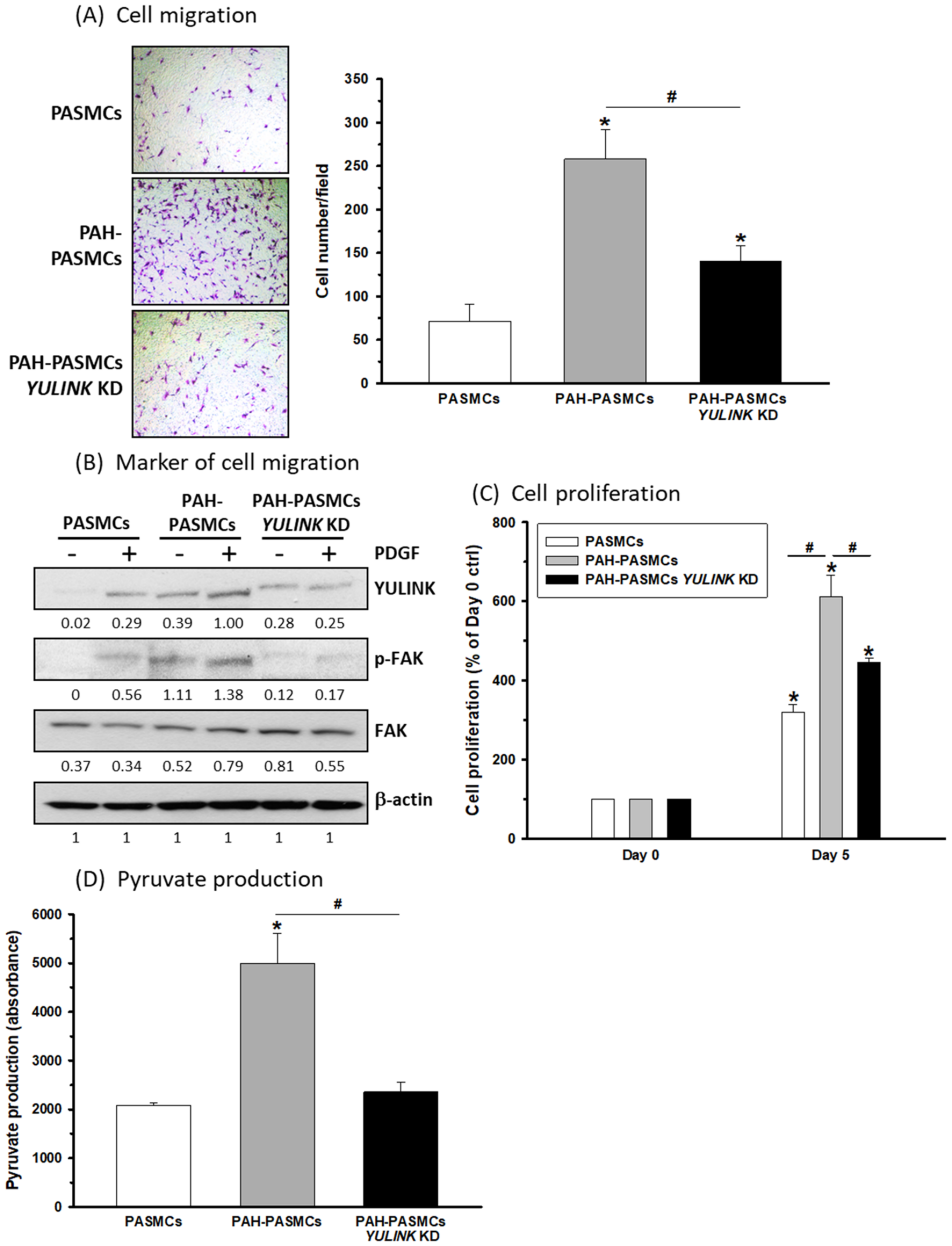
YULINK knockdown reversed the enhancement of cell migration and proliferation in PAH-PASMCs. PASMCs and cells derived from MCT-induced PAH rats with or without *YULINK* KD were prepared for Transwell cell migration analysis. **A** Microscopy images presented with bar graphs indicate cell migration in PASMCs. Magnification ×200. **B** The cells were incubated with PDGF (20 ng/ml) for 24 h before harvest for protein extractions. Western blot analysis indicates the activation and expression of the indicated proteins. β-actin served as an internal control. The numbers labeled below the respective blot lanes indicate the relative fold normalized with the internal control. **C** MTT analysis of the 5-day proliferation of the cells. **D** The production of pyruvate in the cells. The values represent the mean of three independent experiments ± standard deviation. *p < 0.05 compared to normal PASMCs. ^#^p < 0.05 between compared groups

To investigate whether cell migration and proliferation could be correlated with YULINK expression in PAH-PASMCs, cells with or without *YULINK* knockdown were analyzed by Transwell migration and proliferation assays. In comparison with PASMCs derived from normal rats, PAH-PASMCs exhibited a four- to fivefold increase in cell migration (Fig. 5A). However, when *YULINK* was knocked down, the cell migration was dramatically reduced by half in PAH-PASMCs (Fig. 5A); furthermore, as shown in Fig. 5B, increased expression of YULINK and FAK phosphorylation were noted in PAH-PASMCs compared with PASMCs and were further enhanced by PDGF treatment in both PASMCs and PAH-PASMCs, although both were inhibited in PAH-PASMCs with *YULINK* knockdown.

Consistently, cell proliferation was enhanced by 50% in a 5-day proliferation analysis in PAH-PASMCs compared to PASMCs; however, cell proliferation was inhibited in PAH-PASMCs with *YULINK* knockdown (Fig. 5C). As shown in Fig. 5D, pyruvate production increased in PAH-PASMCs, which indicated increased glycolysis compared to that in PASMCs, whereas it decreased in PAH-PASMCs with *YULINK* knockdown compared to that in PASMCs. Taken together, PAH-PASMCs revealed increased cell migration, proliferation and glycolysis compared to PASMCs, which could be associated with YULINK expression.

### YULINK participated in cell migration and proliferation of PASMCs via the PI3K-AKT signaling pathway

**Fig. 6:**
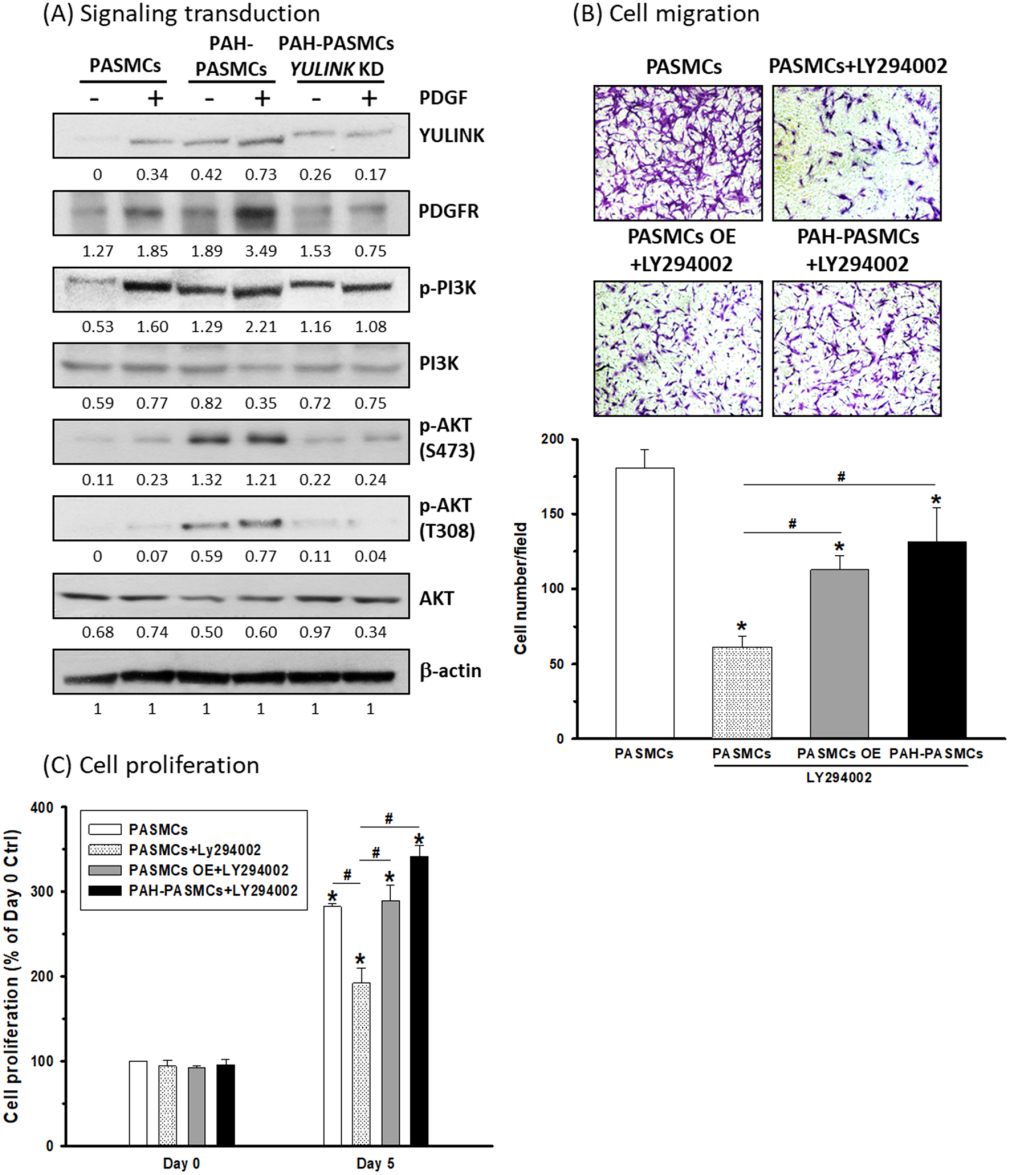
YULINK regulated cell migration and proliferation in PASMCs via PI3K-AKT signaling. **A** PASMCs and PAH-PASMCs with or without *YULINK* KD were treated with or without 20 ng/ml PDGF for 24 h for protein extraction. Cell lysates were subjected to western blot analysis for the indicated proteins. β-Actin served as an internal control. The numbers labeled below the respective blot lanes indicate the relative fold normalized with the internal control. **B** Representative images (magnification 200×) together with the bar graphs indicate the cell migration analysis of PASMCs, PASMCs with or without *YULINK* OE treated with LY294002 (10 µM), and PAH-PASMCs treated with LY294002. **C** A 5-day cell proliferation assay in the cells under the indicated treatments. *p < 0.05 compared to control PASMCs. ^#^p < 0.05 between compared groups

The phosphoinositide 3-kinase (PI3K)-protein kinase B (AKT) signaling pathway is one of the major downstream effects of the PDGF signaling pathway [23] and has been demonstrated to play an essential role in PDGF-induced PASMC proliferation, migration, and PAH progression [8, 24]. To explore the role of YULINK and its potential mechanism in PAH-related cell migration and proliferation, PASMCs derived from normal or MCT-induced PAH rats with or without *YULINK* knockdown were treated with PDGF for further experiments. As shown in Fig. 6A, the expression of YULINK and PDGFR was enhanced in PASMCs and PAH-PASMCs under PDGF treatment. Increased PI3K and AKT phosphorylation in response to PDGF treatment was found in the western blotting analysis, which indicated that PI3K-AKT signaling was activated in PASMCs and PAH-PASMCs under PDGF treatment. When YULINK was suppressed in PAH-PASMCs, the expression of PDGFR and the phosphorylation of PI3K and AKT were both inhibited with or without PDGF treatment.

To further examine whether YULINK could be involved in PASMC migration and proliferation via PI3K-AKT signaling, a PI3K inhibitor, LY294002, was administered to PASMCs with or without *YULINK* overexpression and PAH-PASMCs for further analysis. As shown in Fig. 6B, while treatment with LY294002 alone significantly reduced cell migration in PASMCs, the cell migration ability was rescued in PASMCs with *YULINK* overexpression under LY294002 treatment. In Fig. 6C, similar results were found in the cell proliferation experiment, with a 5-day proliferation assay showing significant inhibition of PASMC proliferation under LY294002 treatment. Furthermore, the downregulated cell proliferation induced by LY294002 treatment was restored in PASMCs overexpressing *YULINK*.

Taken together, the cell migration and proliferation of PASMCs under PDGF treatment or PAH-PASMCs might be regulated by YULINK through the PI3K-AKT signaling pathway.

## Discussion

Despite advances in modern medicine, PAH is still a life-threatening and challenging disease, so lung transplantation could be the only solution for patients with end-stage PAH. Three cellular signaling pathways, endothelin, nitric oxide/cyclic guanosine monophosphate (cGMP) signaling and prostacyclin pathways, have been utilized in the current treatment of PAH in such patients [25]. Nonetheless, existing therapeutic strategies like sildenafil [26], one of the most common treatments for PAH in clinical practice, predominantly target symptomatic relief through nitric oxide-induced vasodilation. Although these approaches can temporarily alleviate disease progression and improve quality of life [26], they are not curative and do not significantly impact mortality, particularly in severe cases.

Pulmonary vascular remodeling caused by aberrant cell migration and proliferation of PASMCs is the major pathological feature of PAH [27], so pharmacological approaches targeting cell migration and proliferation have been widely applied as a therapeutic strategy for PAH [28]. For example, the authors recently found that B-type natriuretic peptide inhibits angiotensin II-induced PASMC proliferation and migration via the cGMP/PKG pathway, which might show therapeutic potential for PAH therapy [29]. However, the genetic role for mediating pulmonary vascular remodeling has not been fully elucidated. In this study, we demonstrated that YULINK overexpression enhanced PDGF-induced PASMC migration through FAK activation; similarly, YULINK promoted proliferation and DNA synthesis in the cell cycle of PASMCs. As a result, YULINK might play various roles in the remodeling mechanisms of PAH.

Accelerated glycolysis, a cancer-like glucose metabolic reprogramming, has recently been noted as a unique feature in PAH pathogenesis. Positron emission tomography scans have shown that PDGF uptake is increased in both animal models and patients with PAH [14], so targeting a regulator that participates in glycolysis has been considered a potential promising approach in PAH therapeutics. The present study demonstrated that YULINK was involved in glycolysis, including glucose uptake, GLUT1 expression, HK-2 expression and pyruvate production, through GLUT1 interaction; additionally, the expression of HK-2 in PASMCs was decreased when YULINK was suppressed. Among the four isoforms of hexokinase, studies have revealed that the activity of HK-2 is significantly increased in rapidly growing cells compared with normal cells and plays a first rate-limiting role in glycolysis for energy supply in abnormally proliferating cells [27, 28]. Our results are in line with a previous study targeting HK-2 by using the noncoding microRNA miR125a-5p to inhibit glycolysis as an approach to improve PAH [29]. Therefore, YULINK could be a promising target for glycolysis in the management of PAH.

This study demonstrated that enhanced YULINK expression was found in PDGF-treated PASMCs, PASMCs and pulmonary arteries of MCT-induced PAH rats, and the pulmonary arteries of a patient specimen. PDGF and its receptor PDGFR have been reported to coordinately regulate pulmonary vascular remodeling by mediating cell proliferation and migration, which are key mechanisms mediating PAH pathogenesis [30]. Therefore, targeting PDGFR by receptor tyrosine kinase inhibitors such as imatinib is considered a new intriguing strategy to treat PAH [31]. Administering imatinib not only reversed experimental pulmonary hypertension in an animal disease model but also improved outcomes in patients with end-stage PAH [32]. In our results, suppressing YULINK significantly reversed cell migration and proliferation of PASMCs not only in PDGF treatment but also in MCT-induced PAH rats. Our findings suggest that YULINK may serve as another attractive target in PAH-targeted therapy.

Furthermore, published reports support the importance of PI3K-AKT in pulmonary vascular remodeling and the pathological development of PAH. The PI3K-AKT pathway is another signaling cascade that has been well demonstrated to contribute to cell migration and proliferation in various cell types, including PASMCs, in PAH [30]. PI3K-AKT inhibition using a small molecular inhibitor, LY294002, can reverse hypoxia-induced anti-apoptosis and PASMC proliferation under hypoxic conditions [31–33]. Pharmacological experiments using multi-kinase inhibitors such as sorafenib in animal models also showed therapeutic benefits in reversing the development of MCT-induced PAH [12]. Knockout of AKT1 in mice was also demonstrated to attenuate vascular remodeling and exhibited a protective effect against the development of hypoxia-induced pulmonary hypertension [34]. Furthermore, the PI3K-AKT pathway has been reported to be involved in glucose metabolism, particularly in regulating GLUT1 and hexokinase 2 to mediate glycolysis in various types of cancer [35–37]. This pathway has also been shown to promote the Warburg effect in PASMCs in response to PDGF stimulation [8]. Although further investigation is required, it may exhibit similar regulatory mechanisms in PAH pathogenesis due to the shared characteristics between cancer and PAH development.

## Conclusions

**Fig. 7:**
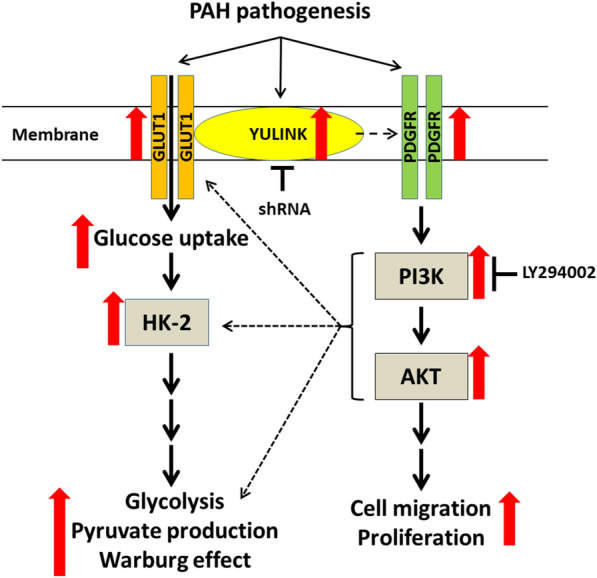
Putative roles of YULINK in PDGF- or MCT-induced PAH pathological aberrations. During PAH-related pathogenesis induced by PDGF or MCT, an increase in YULINK expression occurs. YULINK coordinates with GLUT1 to facilitate glucose uptake, accompanied by an upregulation of HK-2, promoting enhanced glycolysis in PASMCs. This increased YULINK expression also contributes to uncontrolled cell migration and proliferation through the PDGFR-PI3K-AKT signaling pathways. While numerous reports have highlighted the role of this pathway in glucose metabolism and the Warburg effect, whether the triggered PI3K-AKT signaling regulates PAH-related processes requires further investigation. Suppression of YULINK through gene knockdown or the use of an inhibitor LY294002 to suppress PI3K activation has the potential to reverse these pathological abnormalities associated with PAH. In this graph, the black arrows represent the signaling transduction pathways, and the red arrows denote the enhancements in response to PAH pathogenesis. The dotted lines summarize findings from other published literature, not experiments conducted in this manuscript

Our results demonstrate that YULINK inhibition decreased the expression of PDGFR or the activation of PI3K-AKT signaling in PASMCs with PDGF treatment or PAH-PASMCs; furthermore, the inhibition of cell migration and proliferation by LY294002 treatment was significantly reversed in PASMCs with YULINK overexpression or PAH-PASMCs, so the PDGF/PI3K/AKT axis is involved in the effects of YULINK on the pathophysiological development of PAH. Since aberrant proliferation and migration of PASMC, and dysregulated glucose metabolism are the major reasons for PAH, our study indicates that YULINK plays a crucial role in the migration, proliferation and glycolysis of PASMCs, as shown in Fig. 7. Consequently, targeting YULINK holds promise as a potential therapeutic strategy for the treatment of PAH. Further animal and clinical studies are needed to substantiate these important findings.

### Supplementary Information

**Additional file 1: Figure S1.** Colony formation analysis. A total of 3 × 10^2^ PASMCs, with their respective treatments as indicated, were seeded into six-well plates and allowed to grow for 10 days. The formed colonies were then fixed with 4% paraformaldehyde and stained with crystal violet. **Figure S2.** Morphological changes of pulmonary artery in MCT-induced PAH rats. Tissues of pulmonary artery were derived from normal and MCT-induced PAH rats respectively, for Hematoxylin & Eosin staining. Representative photomicrographs indicate the MCT-induced morphological changes in the tissues of pulmonary artery. Magnification 200×.

## Data Availability

Not applicable.

## Abbreviations

PASMCs: Pulmonary arterial smooth muscle cells
PAH: Pulmonary arterial hypertension
Mios: Meiosis Regulator for Oocyte Development
PDGF: Platelet-derived growth factor
DMEM: Dulbecco’s modified Eagle’s medium
FBS: Fetal bovine serum
PBS: P hosphate-buffered saline
ANOVA: Analysis of variance
PDGFR: Platelet-derived growth factor receptor
MCT: Monocrotaline
GLUT1: Glucose transporter 1
HK-2: Hexokinase II
mPAP: Mean pulmonary artery pressure
mTOR: Mammalian target of rapamycin
PI3K: Phosphoinositide 3-kinase
KD: Knockdown
PI: Propidium iodide
2-DG: 2-Deoxyglucose
Ex/Em: Excitation/emission
eNAMPT: Extracellular nicotinamide phosphoribosyltransferase
HMGB-1: High mobility group box 1
