# Supplementary material for: The novel roles of YULINK in the migration, proliferation and glycolysis of pulmonary arterial smooth muscle cells: implications for pulmonary arterial hypertension
Source: Biol Res. 2023 Dec 7;56:66. doi: 10.1186/s40659-023-00480-z (PMC10702011; doi:10.1186/s40659-023-00480-z)
Supplement: Supplementary file 1 — Additional file 1: Figure S1. Colony formation analysis. A total of 3 × 10^2^ PASMCs, with their respective treatments as indicated, were seeded into six-well plates and allowed to grow for 10 days. The formed colonies were then fixed with 4% paraformaldehyde and stained with crystal violet. Figure S2. Morphological changes of pulmonary artery in MCT-induced PAH rats. Tissues of pulmonary artery were derived from normal and MCT-induced PAH rats respectively, for Hematoxylin & Eosin staining. Representative photomicrographs indicate the MCT-induced morphological changes in the tissues of pulmonary artery. Magnification 200×. [file 40659_2023_480_MOESM1_ESM.docx]

**Figure S1**


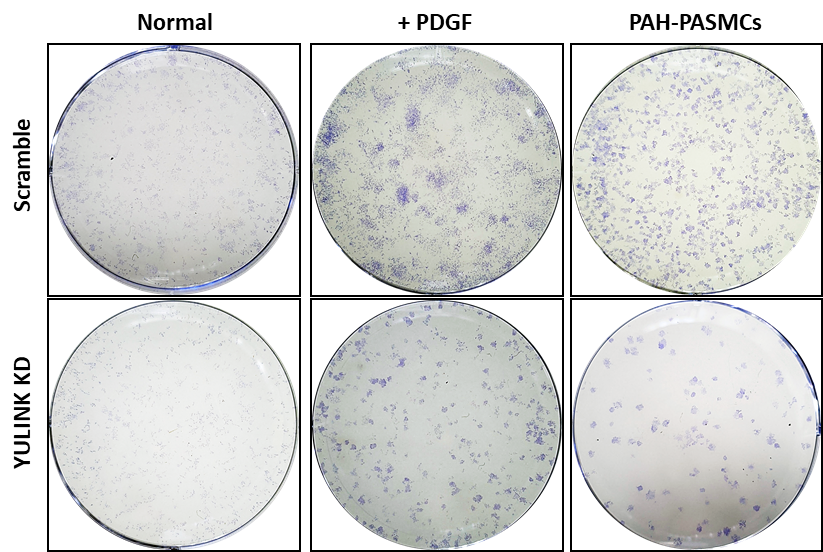


**Fig. S1. Colony formation analysis.** A total of 3×10^2^ PASMCs, with their respective treatments as indicated, were seeded into six-well plates and allowed to grow for 10 days. The formed colonies were then fixed with 4% paraformaldehyde and stained with crystal violet.

**Figure S2.**


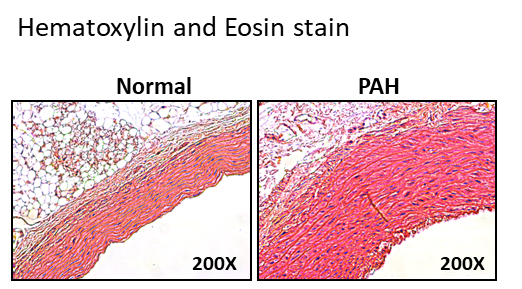


**Fig. S2. Morphological changes of pulmonary artery in MCT-induced PAH rats.** Tissues of pulmonary artery were derived from normal and MCT-induced PAH rats respectively, for Hematoxylin & Eosin staining. Representative photomicrographs indicate the MCT-induced morphological changes in the tissues of pulmonary artery. Magnification 200×.
